# SLC2A3 promotes macrophage infiltration by glycolysis reprogramming in gastric cancer

**DOI:** 10.1186/s12935-020-01599-9

**Published:** 2020-10-12

**Authors:** Xingxing Yao, Zhanke He, Caolitao Qin, Xiangqian Deng, Lan Bai, Guoxin Li, Jiaolong Shi

**Affiliations:** 1 Department of General Surgery, Guangdong Provincial Engineering Technology Research Center of Minimally Invasive Surgery, Nanfang Hospital, Southern Medical University, Guangzhou, Guangdong China; 2 Guangdong Provincial Key Laboratory of Gastroenterology, Department of Gastroenterology, Nanfang Hospital, Southern Medical University, Guangzhou, 510515 Guangdong China

**Keywords:** SLC2A3, Glycolysis reprogramming, Macrophage, Tumor microenvironment, Gastric cancer

## Abstract

**Background:**

Tumors display a high rate of glucose metabolism and the SLC2A (also known as GLUT) gene family may be central regulators of cellular glucose uptake. However, roles of SLC2A family in mechanism of metabolite communication with immunity in gastric cancer remains unknown.

**Methods:**

Bioinformatics analysis and IHC staining were used to reveal the expression of SLC2A3 in gastric cancer and the correlation with survival prognosis. Real-time PCR, western blots, OCR, ECAR, lactate production and glucose uptake assays were applied to determine the effect of SLC2A3 on glycolysis reprogramming. We then investigated the consequences of SLC2A3 upregulation or inhibition on aerobic glycolysis, also explored the underlying mechanism. Bioinformatics analysis and in vitro and in vivo research were used to reveal the role of SLC2A3 in macrophage infiltration and transition.

**Results:**

Here, we show that SLC2A3 acts as a tumor promoter and accelerates aerobic glycolysis in GC cells. Mechanistically, the SLC2A3-STAT3-SLC2A3 feedback loop could promote phosphorylation of the STAT3 signaling pathway and downstream glycolytic targeting genes. Moreover, SLC2A3 potentially contributes to M2 subtype transition of macrophage infiltration in the GC microenvironment.

**Conclusions:**

SLC2A3 could be used as a prognostic biomarker to determine prognosis and immune infiltration in GC and may provide an intervention strategy for GC therapy.

## Background

Gastric cancer (GC) is the fourth most common cancer and the second leading cause of cancer-related death worldwide [1]. With symptoms that are often mistaken for other gastric diseases, many patients with GC are diagnosed when the cancer is already at an inoperable or metastatic stage [2]. Treatment options for advanced GC are limited, while cancer progression is generally aggressive and treatment response is poor [2]. A few biomarkers have been used as therapeutic targets for advanced GC [3]. However, therapeutic outcomes remain unsatisfactory, which may be due to multiple genetic variations and changes in the microenvironment, such as altered glucose metabolism promoting gastric carcinogenesis [4].

Tumor displays high metabolic rate, high glucose requirement, and increased glucose uptake [5]. As the concentrations of highly consumed nutrients, particularly glucose, are generally lower in tumors than in normal tissues, cancer cells must adapt their metabolism to the tumor microenvironment (TME) [6]. In mammalian cells, glucose transport across the plasma membrane is mediated by solute carrier 2A (SLC2A) family (also referenced as the glucose transporter, or GLUT family) [7]. The SLC2A protein family consists of fourteen 12-transmembrane domain-containing proteins that catalyze facilitative diffusion of glucose and other monosaccharides, thus, these carrier proteins are central regulators of cellular energetics [8]. High sugar transport facilitator expression levels contribute to augmented glucose uptake and oncogenic growth [8]. An association between the overexpression of a subtype of SLC2A proteins and poor clinical outcomes has been reported in colorectal cancer [9, 10]. In patients with non-small-cell lung cancer, SLC2A1 expression is related to poor prognosis [11, 12]. Kuang et al. revealed comprehensive metabolic reprogramming in tumors resistant to antiangiogenic therapy emanating from increased SLC2A3 expression [13]. Additionally, a previous study found that high SLC2A1 expression is involved in low CD8(+) T-cell infiltration in renal cancer. These findings suggest that SLC2A proteins have potential functional roles in regulating tumor-infiltrating immune cells [14]. However, whether SLC2A proteins affect the TME in GC has not yet been elucidated.

The TME consists of a complex mixture of malignantly transformed cells, stromal cells, and immune cells that fulfill different functions [15]. One of the most abundant components of the TME are tumor-associated macrophages (TAMs), which contribute to tumor progression at several levels, including promoting genetic instability, nurturing cancer stem cells, paving the way to metastasis, and taming protective adaptive immunity [16]. Recent studies have suggested that TAMs can be functionally categorized into tumor-supportive (M2 type) macrophages and tumor-suppressive (M1 type) macrophages [17]. TAMs are relatively skewed to the M2 type, contributing to the malignant phenotype and immunoregulation [18]. More recently, it has become clear that the heterogeneity of macrophage populations depends on the cues that they are exposed to [19]. In response to tumor cell-derived metabolic products, TAMs undergo metabolic reprogramming which subsequently influences their functional phenotype [15]. Additionally, GC cells have extensively reprogrammed metabolism, driven by the unique physiology of the TME, and interactions with macrophages. Schlo¨ßer et al. reported that SLC2A3 is expressed in a significant proportion of GC samples and is associated with a poorer prognosis [20]. However, the underlying functions and mechanisms of SLC2A proteins in GC progression and tumor immunology remain unclear.

This study was designed to identify mechanisms mediating the cancer-promoting effects of SLC2A3 in GC, with focus on its glycolysis reprogramming function, and to reveal molecular links between SLC2A3 and immune regulation.

## Results

### SLC2A3 expression is an independent risk factor and leads to a poor prognosis in GC patients

**Fig. 1:**
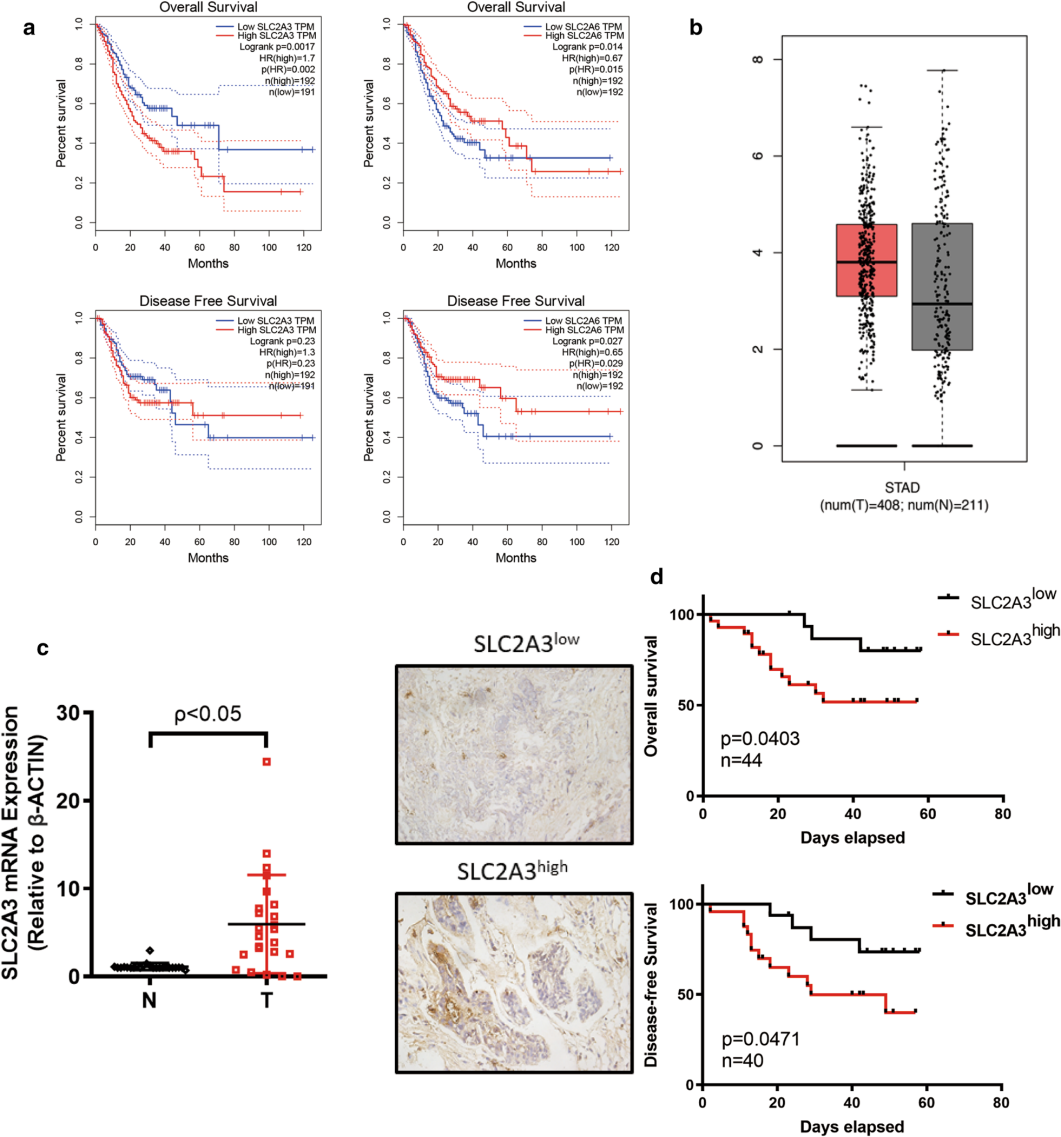
SLC2A3 overexpression correlated with poor prognosis in gastric cancer (GC). **a** Overall survival (OS) curves (n (high) = 192, n (low) = 191) and disease-free survival (DFS) survival curves for patients with GC by SLC2A3 expression (n (high) = 192, n (low) = 191). OS survival curves (n (high) = 192, n (low) = 192) and DFS survival curves for patients with GC by SLC2A6 expression (n (high) = 192, n (low) = 192). **b** SLC2A3 mRNA expression in stomach adenocarcinoma (GEPIA database). **c** SLC2A3 mRNA expression in GC tissue and paired normal gastric mucosa as determined by RT-PCR. **d** SLC2A3 IHC staining in GC samples (representative images), OS survival curve (n = 44), and DFS survival curve of SLC2A3 high and low groups in GC (n = 40)

To explore the clinical relevance of the SLC2A family in GC, we analyzed the relationship between expression of SLC2A members and disease-free survival (DFS) and overall survival (OS) in patients with GC using the Gene Expression Profiling Interactive Analysis (GEPIA) database. Notably, we found that higher SLC2A3 expression positively correlated with poorer OS (Fig. 1a, p = 0.002, n (high) = 192, n (low) = 191) in patients with GC. SLC2A6 expression was also positively correlated with poorer prognosis (Fig. 1a, p = 0.015, n (high) = 192, n (low) = 192) and DFS (Fig. 1a, p < 0.029, n (high) = 192, n (low) = 192). Conversely, higher SLC2A3 expression in GC was not significantly related to DFS (Fig. 1a, p = 0.23, n (high) = 192, n (low) = 191). Other family members did not show any significant correlation with survival (Additional file 1: Figure S1). Therefore, high SLC2A3 and SLC2A6 expression are potential risk factors leading to poor prognosis in patients with GC. Previously, it was described that SLC2A6 was expressed in low levels in GC while the SLC2A3 expression was linked to clinical and pathological parameters [21]. Therefore, we selected SLC2A3 as a target of interest in our following research. To further evaluate SLC2A3 expression in tumorigenesis, we examined SLC2A3 expression using the RNA-seq data from multiple malignancies in The Cancer Genome Atlas (TCGA). Differential SLC2A3 expression between tumor and adjacent normal tissues across all tumors is shown in Additional file 1: Figure S2. We found that SLC2A3 was more highly expressed in GC than normal tissues but that this difference was not significant (Fig. 1b). However, mRNA analysis using our center’s data base showed that SLC2A3 expression was higher in GC tumors than in paired normal mucosa (n = 32, p < 0.05, Fig. 1c). Next, we divided clinical patients into SLC2A3^low^ and SLC2A3^high^ groups based on their immunohistochemistry (IHC)-paraffin staining score ranking. The clinicopathological parameters of the 44 patients with GC included in the OS analysis are displayed in Table 1 and those of the 40 patients with GC included in the DFS analysis are displayed in Table 2. Correlation analysis results showed that higher SLC2A3 expression predicted worse DFS and OS (p_OS_ = 0.0403, n_OS_ = 44; p_DFS_ = 0.047, n = 40) (Fig. 1d). Collectively, these results suggest that SLC2A3 is upregulated in GC and that high SLC2A3 expression leads to an unfavorable prognosis for patients with GC.

**Table 1:** The association between SLC2A3 mRNA levels and the clinicopathological parameters in 44 GC patients included in the OS analysis

Clinicopathological parameters	SLC2A3 expression		p-value
	Low	High	
Age			
< 60	8	13	p = 0.533
≥ 60	8	15	
Gender			
Male	12	18	p = 0.350
Female	4	10	
pStage			
I	5	2	p = 0.049
II–IV	11	26	
pT grade			
T1, 2	7	4	p = 0.037
T3, 4	9	24	
pN grade			
N0	9	10	p = 0.157
N1–3	7	18	
pM grade			
M0	16	26	p = 0.400
M1	0	2	
Total	16	28	

SLC2A3 protein expression was significantly higher in patients with pathological stage (p = 0.049) and advanced T (p = 0.037) but showed no association with age, gender, N and M grades

*GC* Gastric cancer, *OS* overall survival, *TNM* tumor, *N* lymph node, *M* metastasis

**Table 2:** The association between SLC2A3 protein levels and the clinicopathological parameters in 40 GC patients included in the DFS analysis

Clinicopathological parameters	SLC2A3 expression		p-value
	Low	High	
Age			
< 60	8	10	p = 0.422
≥ 60	8	14	
Gender			
Male	12	18	p = 0.649
Female	4	6	
pStage			
I	5	2	p = 0.076
II–IV	11	22	
pT grade			
T1, 2	7	4	p = 0.065
T3, 4	9	20	
pN grade			
N0	9	10	p = 0.281
N1–3	7	14	
Total	16	24	

SLC2A3 protein expression was showed no association with age, gender, pathological stage, and TNM grades

*GC* Gastric cancer, *OS* overall survival, *TNM* tumor, *N* lymph node, *M* metastasis

### SLC2A3 mediates gastric cancer glycolysis reprogramming

**Fig. 2:**
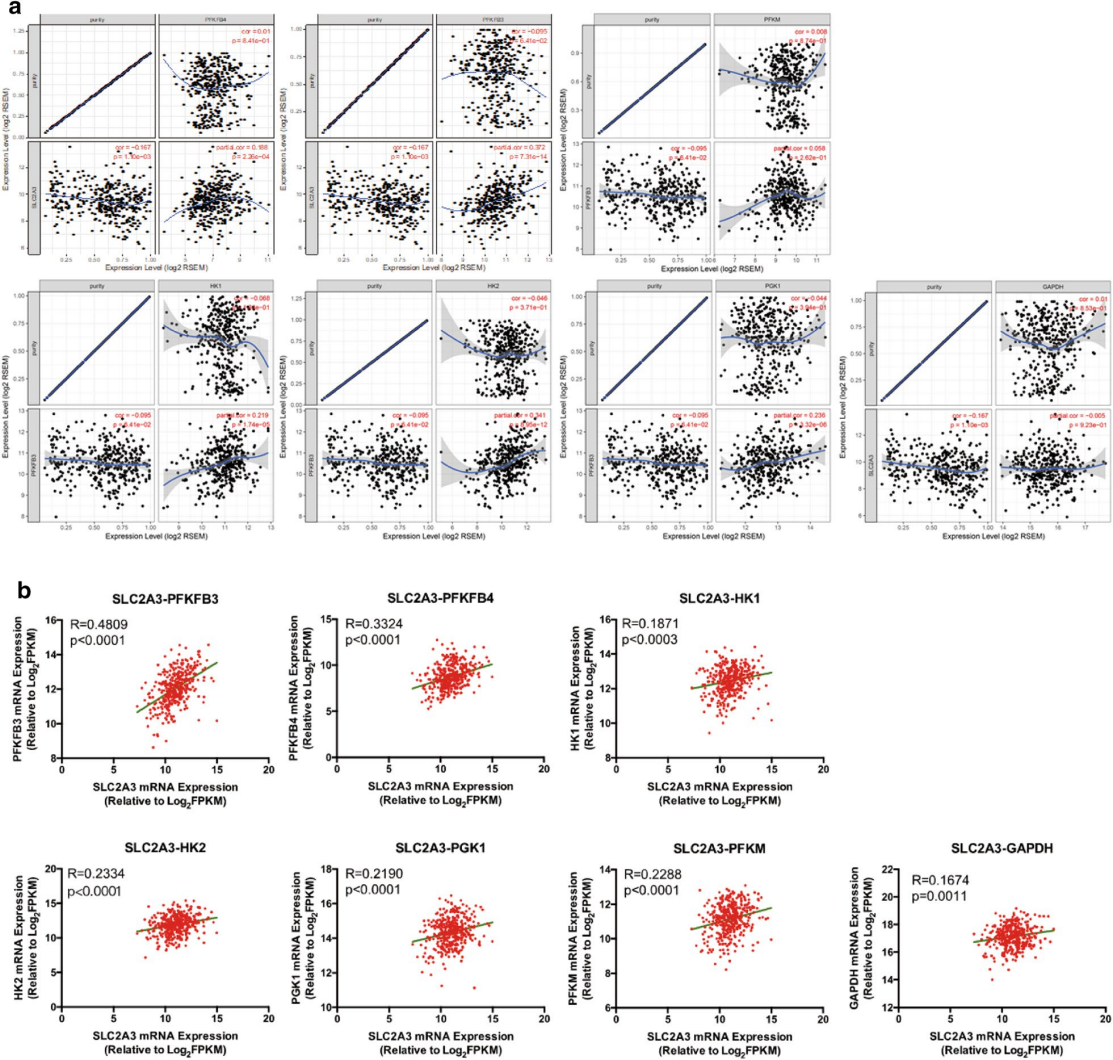
SLC2A3 promoted the expression of glycolysis enzymes expression in GC. **a** The relationship between SLC2A3 and PFKFB3, PFKFB4, HK1, HK2, PGK1, PFKM, and GAPDH as revealed by TIMER database analysis. **b** The relationship between the SLC2A3 mRNA levels and those of PFKFB3, PFKFB4, HK1, HK2, PGK1, PFKM, and GAPDH as revealed by linear regression analysis

Considering that SLC2A3 protein control glycolytic flux through glycolysis [22], we explored the function of SLC2A3 in regulating glycolysis. The TIMER database revealed that SLC2A3 expression was significantly positively correlated with of PFKFB3, PFKFB4, HK1, HK2, and PGK1 expression (Fig. 2a). Consistent with data in TIMER, the TCGA database also revealed that SLC2A3 over-expression was positively correlated with upregulation of multiple glucose transporter, phosphofructokinases, and pyruvate kinases isozymes (Fig. 2b). These results indicate that SLC2A3 could mediate GC glycolysis reprogramming.

### SLC2A3 accelerated gastric cancer proliferation by activating glycolysis reprogramming

**Fig. 3:**
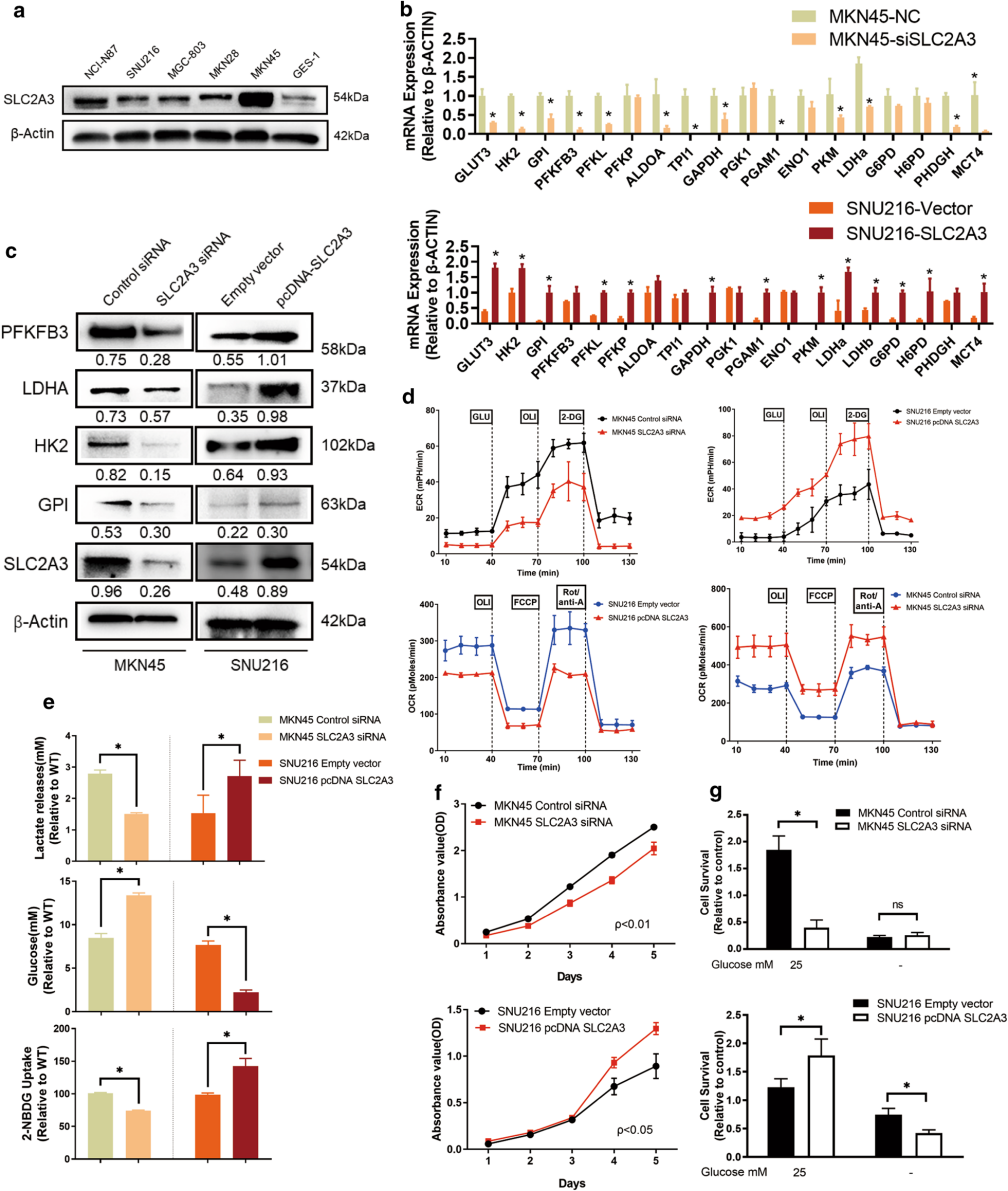
SLC2A3 activates GC cell proliferation by activating glycolysis reprogramming. **a** SLC2A3 protein expression was detected in GES-1 cells and in seven different gastric cancer cell lines. **b** RT-PCR was used to detect the expression levels of glycolysis enzymes in SLC2A3 overexpression and knockdown cells. **c** Changes in protein levels were detected by western blot analysis after transfection with SLC2A3 siRNA or SLC2A3 overexpression plasmid. **d** MKN45 cells transfected with control and SLC2A3 siRNA, or SNU216 cells transfected with empty vector and SLC2A3 overexpression plasmid, were seeded in 24-well plates and exposed to glucose, oligomycin A, 2-DG FCCP, and rotenone to measure extracellular acidification rates (ECAR) and increased oxygen consumption rates (OCR). **e** Relative lactate release, glucose uptake, and residue glucose from cells was determined by colorimetric analysis. **f** CCK8 assays were used to assess cell proliferation after siSLC2A3 or SLC2A3 overexpression plasmid transfection. **g** The effect of glucose deprivation on the growth of cells with overexpression or knock-down of SLC2A3. Cells were cultured in normal and low glucose (0.5 mM) conditions for 48 h, and then subjected to MTT assays. Relative survival was plotted as the percent of cells cultured in normal glucose. Each experiment was performed at least in triplicate and results are presented as mean ± SD. p-values were calculated by one-way ANOVA followed by SNK multiple comparison test. *p < 0.05

We further compared the SLC2A3 expression levels in seven GC cell lines with normal gastric epithelium cell line GES-1, which is non-malignant and none-tumourigenic cell line. A significant increase in SLC2A3 protein expression was observed in all GC cells compared with GES-1. Furthermore, western blot analysis indicated that SLC2A3 was highly expressed in GC cell lines including MKN45 and MKN28 and expressed at relatively low levels in SNU216 and SNU5 cells (Fig. 3a). Therefore, we selected MKN45 and SNU216 cell lines for the following functional experiments. To investigate the biological behaviors of SLC2A3 in glycolysis process, we knocked-down and upregulated SLC2A3 expression in MKN45 and SNU216 cells, respectively. Then we performed other GLUT isoforms and HMIT assessment by RT-PCR for the first time. Results showed that effects of SLC2A3 perturbation on other GLUT isoforms and HMIT were not obvious (Additional file 1: Figure S3b). Subsequently, RT-PCR and western blot results showed that the expression of most glycolysis-related enzymes decreased significantly following SLC2A3 knockdown and that their expression increased when SLC2A3 was upregulated (Fig. 3b, c). Compared with the control, cells transfected with siSLC2A3 had decreased extracellular acidification rates (ECAR) and increased oxygen consumption rates (OCR). Upregulation of SLC2A3 results in increased ECAR and decreased OCR (Fig. 3d). We also observed significantly decreased lactate release, 2-NBDG uptake, and increased residual glucose in the supernatant of SLC2A3 knockdown cells compared with negative controls. Moreover, glycolytic process was significantly augmented in cells transfected with the SLC2A3 overexpression plasmid (Fig. 3e). SLC2A3 knockdown in MKN45 cells significantly inhibited cell growth, while SLC2A3 upregulation promoted cell proliferation in SNU216 cells (Fig. 3f). To determine the phenotypic change in glucose metabolism led by SLC2A3, we cultured the control cells and corresponding SLC2A3 knockdown or upregulated cells under low glucose conditions (0.5 mM glucose) for a short period (48 h). Cell survival was promoted in MKN45 cells with knocked-down SLC2A3 under low glucose conditions, but the difference was not statistically significant. Meanwhile, SLC2A3-overexpressed SNU216 cells demonstrated significantly diminished survival under low glucose conditions (Fig. 3g). We hypothesized that SLC2A3 overexpression might promote GC proliferation by turning on glycolysis in GC cells.

### The SLC2A3-STAT3-SLC2A3 feedback loop promoted gastric cancer progression

**Fig. 4:**
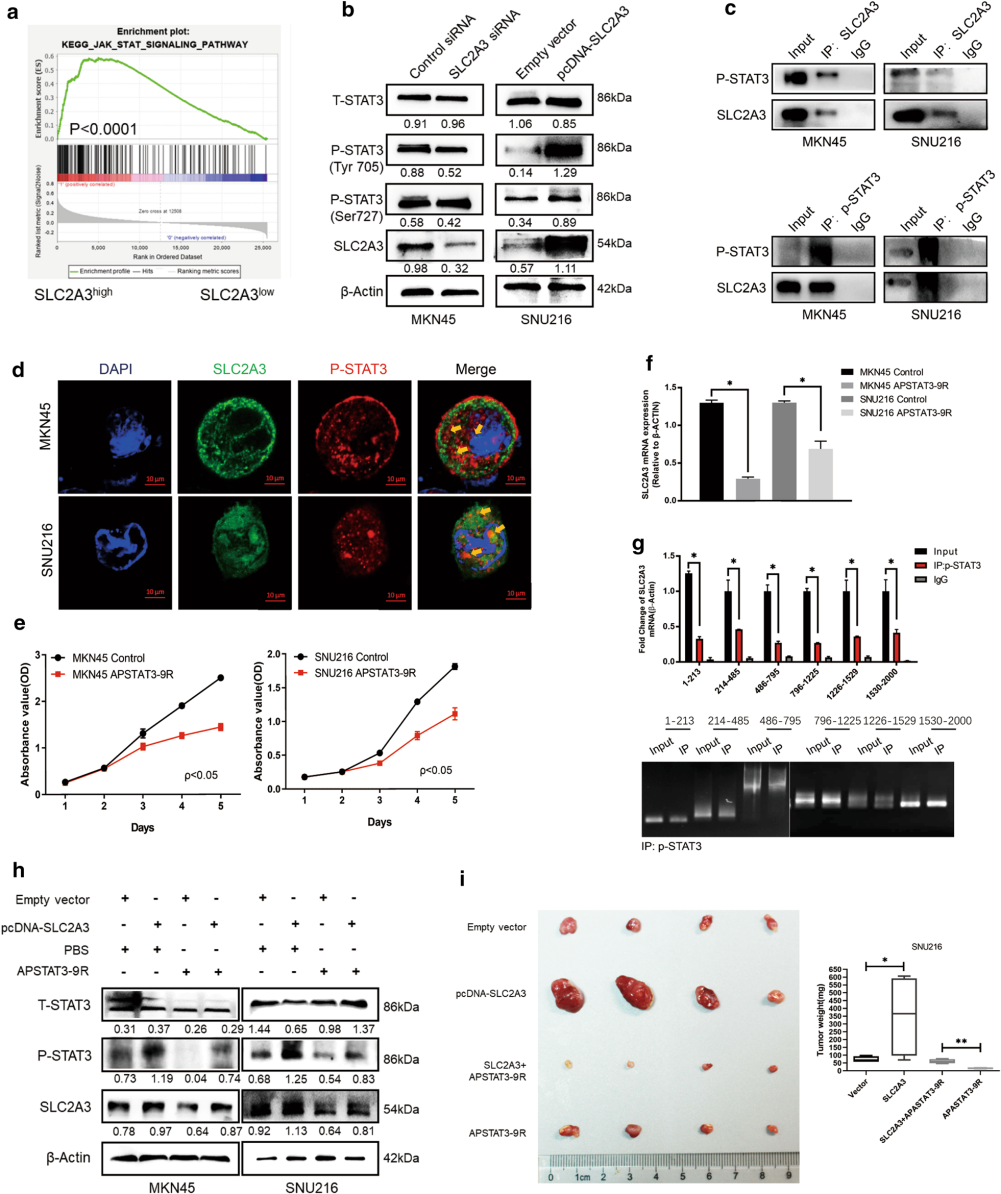
The SLC2A3-STAT3-SLC2A3 feedback loop accelerates GC progression. **a** Gene-set enrichment analysis (GSEA) of the protein profiles between SLC2A3^high^ and SLC2A3^low^ groups in the database. **b** Expression of STAT3 signaling pathway related proteins detected in MKN45 and SNU216 cells by western blot after transfection with siSLC2A3 or SLC2A3 overexpression plasmid. **c** The interaction between SLC2A3 and p-STAT3 was determined by co-immunoprecipitation analysis. **d** Co-localization of SLC2A3 (green) and p-STAT3 (red) were assessed by immunofluorescence staining. Merged images represent overlays of SLC2A3, p-STAT3, and nuclear staining by DAPI (blue). **e** CCK8 assays were conducted to evaluate changes in proliferation ability by APSTAT3-9R stimulation. **f** Changes in mRNA levels were detected by RT-PCR after APSTAT3-9R stimulation. **g** ChIP-qPCR and PCR analysis of β-catenin with SLC2A3 and STAT3 promoter regions. **h** Changes in the expression o of SLC2A3, STAT3, and p-STAT3 protein in SLC2A3 overexpressing and SLC2A3 knockdown cells with or without APSTAT3-9R stimulation. **i** Subcutaneous xenograft tumor formation with SNU216 cells (Control-LV vs. SLC2A3-LV), followed by treatment with intraperitoneal injection of PBS or APSTAT3-9R (5 mg/kg). Statistical results are shown as mean ± SD, *ρ < .05, **ρ < .001, based on two-way ANOVA or Student’s t-test

To further explore the mechanisms underlying the stimulatory effects of SLC2A3 on glycolysis promotion, we analyzed key signaling pathways that could be influenced in SLC2A3 activated cancer cells. Gene-set enrichment analysis of transcriptome profiles showed that the STAT3 signaling pathway was likely activated in these samples (Fig. 4a). Furthermore, western blot analysis was performed to examine total STAT3 and phosphorylated STAT3 (p-STAT3) expression in cells. Results showed that knock-down of SLC2A3 expression dramatically decreased the phosphorylation of STAT3 at Tyr705 in MKN45 cells. Meanwhile, upregulation of SLC2A3 increased the level of p-STAT3 at Tyr705 (Fig. 4b). However, phosphorylation of STAT3 at Ser727, also regulated by SLC2A3, was not significant. Therefore, Tyr705 was selected as a representative site for subsequent studies. We used Co-IP assays to explore protein interactions between SLC2A3 and p-STAT3 in MKN45 and SNU216 cells, and reciprocal CO-IP further showed that SLC2A3 and p-STAT3 interact (Fig. 4c). This was further confirmed by co-localization of SLC2A3 and p-STAT3 in MKN45 and SNU216 cells as detected by immunofluorescence staining (Fig. 4d). We then used pharmacologic approaches to inhibit p-STAT3 expression. As expected, APSTAT3-9R could markedly weaken the proliferative ability of MKN45 and SNU216 cells (Fig. 4e).

To investigate the transcriptional regulatory mechanism of SLC2A3 expression, we used the JASPAR database (https://jaspar.genereg.net). We identified 36 potential binding sites between transcriptional factor STAT3 and SLC2A3. We then selected the ten representative sites shown in Additional file 1: Figure S4. Evaluation of the mRNA levels of SLC2A3 revealed that the p-STAT3 inhibitor APSTAT3-9R significantly suppressed SLC2A3 transcription in both MKN45 and SNU216 cells (Fig. 4f).

The chromatin immunoprecipitation (ChIP) assay was then used to determine whether p-STAT3 bound the SLC2A3 promoter in SNU216 cells. We designed and composed 6 primers according to the potential binding sites of SLC2A3 promoter regions. ChIP-qPCR and PCR analysis showed that p-STAT3 could bind to the promoter regions of SLC2A3 (Fig. 4g).

Rescue experiments showed that treatment with p-STAT3 inhibitor APSTAT3-9R in GC cells overexpressing SLC2A3 significantly restored SLC2A3-mediated promotion of glycolysis reprogramming. Considering our in vitro findings, SLC2A3-expressing recombinant lentivirus (SLC2A3-LV) or control vectors (Control-LV) were used to establish SLC2A3-overexpressed SNU216 cells. Stable SNU216 cells transfected with either SLC2A3-LV or Control-LV were subcutaneously inoculated in nude mice. Mice were then randomly divided into four groups and administered PBS or APSTAT3-9R intraperitoneally every 2 days. Consistent with previous reports, our results show that the APSTAT3-9R p-STAT3 inhibitor significantly inhibited the proliferation of tumors led by SLC2A3 upregulation (Fig. 4i). Taken together, these results suggest that SLC2A3 might promote GC cell proliferation by activating the phosphorylation of the STAT3 signaling pathway and that STAT3 might mediate SLC2A3 expression through binding to SLC2A3 promoter regions.

### SLC2A3 promotes GC progression by inducing macrophage infiltration and M2 subtype transition

**Fig. 5:**
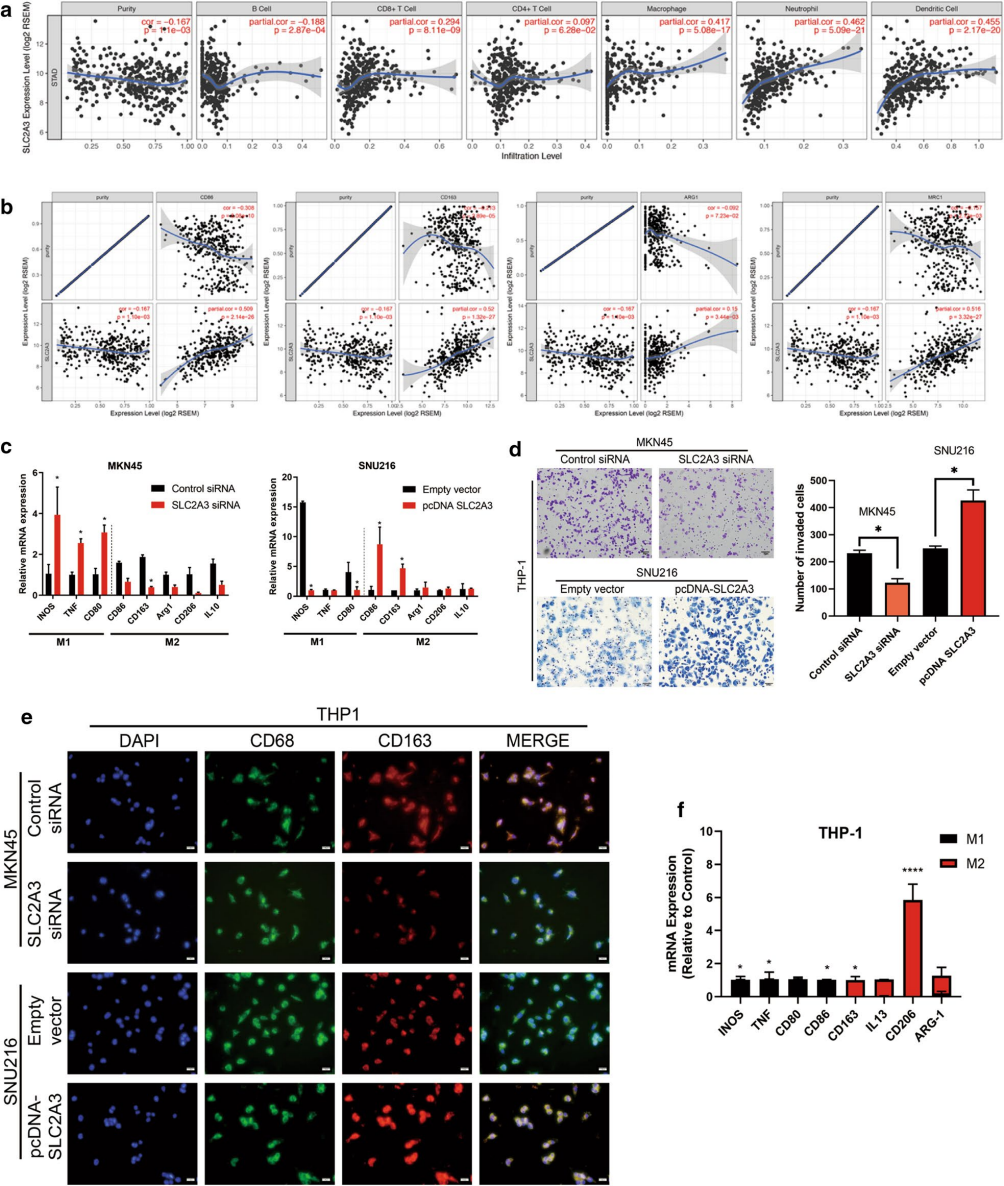
SLC2A3 promotes GC progression by inducing macrophage infiltration and M2 subtype transition. **a** The relationship between SLC2A3 expression and infiltrating levels of B cells, CD8+ T cells, CD4+ T cells, macrophages, neutrophils, and dendritic cells. **b** Scatterplots of the correlations between SLC2A3 expression and macrophage M1 and M2 subtype gene markers obtained using the TIMER database. **c** RT-PCR assays revealed M1 and M2 macrophage subtype marker expression in THP1 cells after co-culture with SLC2A3 overexpressing and knockdown cells. **d** Transwell analysis. **e** THP-1 cells were incubated with supernatant from cells transfected with SLC2A3 overexpression plasmid or siRNA. Cells were then fixed and immunolabeled for CD68 or CD163. Nuclei were stained with DAPI (blue). **f** RT-PCR assay revealed M1 and M2 macrophage subtype marker expression in THP1 cells treated with or without 10 mmol/ml lactate for 24 h. Statistical results are shown as mean ± SD, *ρ < .05, **ρ < .001, ****ρ < .001, based on two-way ANOVA or Student’s t-test

Since TIMER database analysis showed that level of SLC2A3 decreased significantly with improving GC tissue purity and the TME (Fig. 5a), which contains various immune cells, might play an important role in the progression of GC [23]. Therefore, we investigated whether SLC2A3 expression correlated with immune infiltration levels in GC. We assessed the correlation between SLC2A3 expression and the immune infiltration levels from TIMER. The analysis revealed that SLC2A3 expression had significantly positively correlated with the infiltration levels of B cells, CD8+ T cells, CD4+ T cells, macrophages, neutrophils, and dendritic cells infiltration levels in GC (Fig. 5b). Macrophages are one of the most important components of the stroma and shift their functional phenotypes in response to various microenvironmental signals [24]. Therefore, we focused on the correlations between SLC2A3 and macrophage immune marker sets. Our results showed that the CD86 marker of the M1 phenotype, and the CD163, ARG1 and MRC1 markers of the M2 phenotype were significantly correlated with SLC2A3 expression in GC (p < 0.0001) (Fig. 5c). Transwell assays showed that the SLC2A3 knockdown conditional medium could reduce the invasion and migration abilities of THP-1 cells, while SLC2A3 overexpression medium could activate these abilities (Fig. 5d). To determine whether elevated SLC2A3 gene expression induced the inflammatory M2 state, we induced human THP-1 monocytes to differentiate into macrophages using phorbol 12-myristate 13-acetate and then treated with medium conditioned from MKN45 and SNU216 cells. Real-time PCR analysis indicated the expression of that THP-1 monocytes cultured with conditioned medium from SLC2A3 knockdown MKN45 cells had significantly reduced M1 marker (INOS, TNF-a, and CD86) expression and increased M2 marker (CD163, CD206, IL4, and IL13) expression than did the control (Fig. 5e). The opposite results were observed when macrophages were cultured with conditioned medium from SNU216 cells overexpressing SLC2A3. Traditionally, CD68 is exploited as a valuable cytochemical marker to immunostain monocyte/macrophages in the histochemical analysis of inflamed tissues, tumor tissues, and other immunohistopathological applications. Hence, we used CD68 in combination with CD163 (M2 marker) to show tumor-associated macrophages. A similar conclusion was drawn from CD163 (M2 marker) and CD68 (macrophage marker) immunofluorescence assay results. As shown in Fig. 5e, THP-1 were inclined to the M2 phenotype after being cultured with conditioned-medium from SLC2A3 knockdown MKN45 cells. CD163 expression was significantly reduced in THP-1 cells grown in culture medium from SNU216 cells transfected with the SLC2A3 overexpression plasmid. According to the Warburg effect theory, tumor cells predominantly produce energy through high-efficiency glycolysis followed by lactate accumulation, even in the presence of oxygen. To verify that increased lactate production in cells overexpressing SLC2A3 caused of the observed phenotype, we sought to treat THP-1 cells with lactate. The results revealed a significant increase in M2 marker transcript levels and a decrease in M1 marker transcript levels (Fig. 5f).

**Fig. 6:**
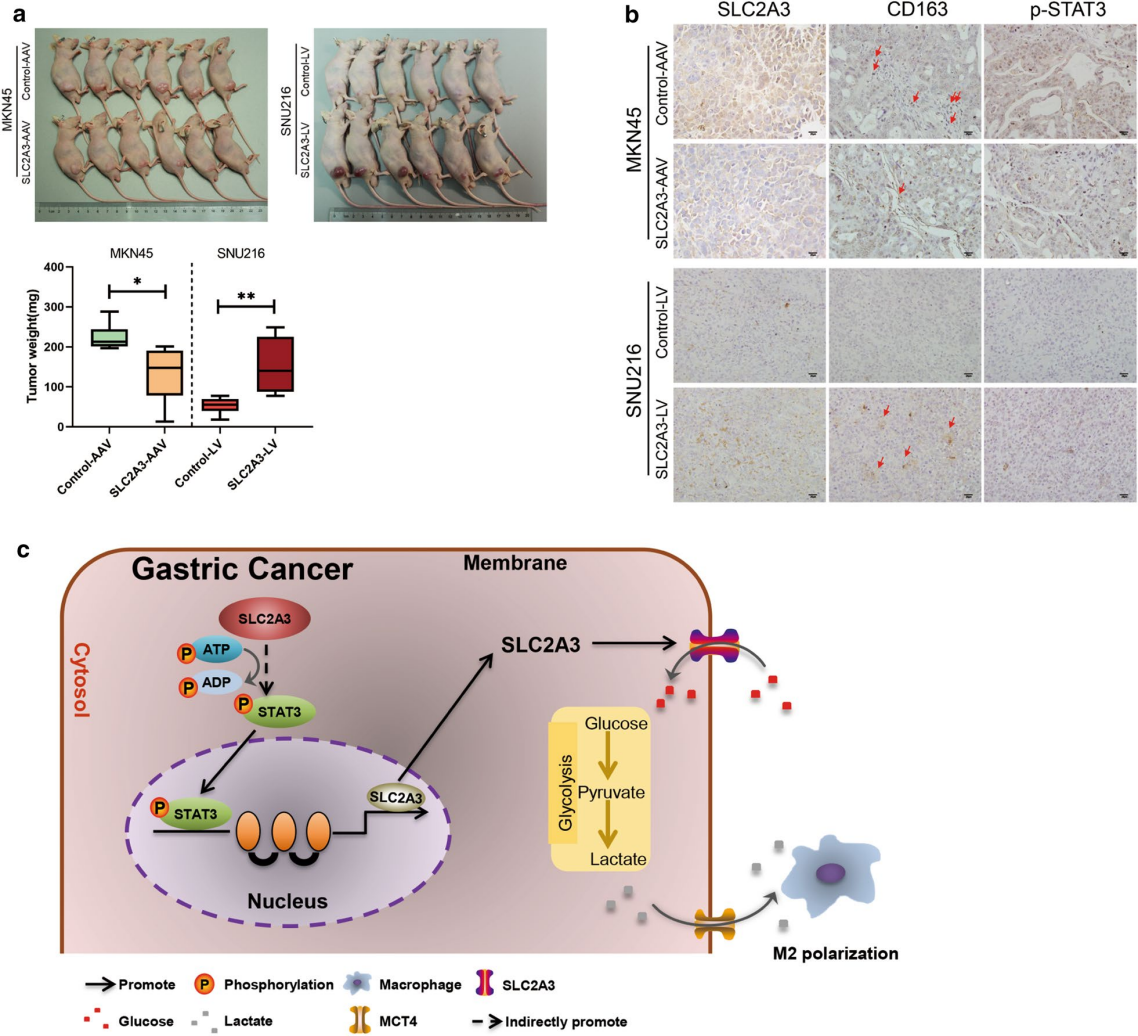
SLC2A3 played tumor-promotive roles in vivo. **a** Sacrificed nude mice treated with SLC2A3-AAV knockdown (AAV-SLC2A3-KD) or control vectors (Control-AAV) and corresponding tumor growth curves. Tumor sizes were measured in triplicate. **b** Representative subcutaneous tumor IHC analysis for SLC2A3, p-STAT3, and CD163 (representative images are shown). **c** The proposed role of SLC2A3 in GC progression. Statistical results are shown as mean ± SD, *ρ < .05, **ρ < .001, based on two-way ANOVA or Student’s t-test

Tissue- or cell-specific promoter regulated recombinant adeno-associated virus vector (AAV) can have a relatively specific tumor targeting effect [25]. We introduced SLC2A3 knockdown and control recombinant AAV-green fluorescence protein vectors into subcutaneous xenograft models to test the functions of SLC2A3 in proliferation in vivo. Results showed that tumors could be detected in both SLC2A3-LV and Control-LV mice and mice in the SLC2A3-LV group had larger tumor volumes than the control mice. Additionally, SLC2A3-AAV (AAV-SLC2A3-KD) markedly suppressed tumor growth compared to negative controls (Fig. 6a). Consistent with in vitro findings, loss of SLC2A3 led significant downregulation of p-STAT3 and decreased M2 macrophage infiltration. The intensity of CD163 and p-STAT3 staining were significantly increased in SLC2A3-LV group than that in the control group (Fig. 6b). Collectively, these results support the contention that SLC2A3 is an important oncogene that changes the GC TME by increasing the M2 macrophage infiltration through releasing lactate.

## Discussion

The solute carrier 2A (SLC2A) gene family that encodes glucose transporter (GLUT) proteins has not been widely investigated. Recently, upregulation of SLC2A genes were reported to be associated with poor prognosis in cancers, including breast cancer [26], non-small cell lung cancer [27], and thyroid carcinoma [26]. Here, we found that SLC2A3 was commonly upregulated in human GC, and that SLC2A3 overexpression was related to poor survival and was an unfavorable prognostic indicator for patients with GC. Interestingly, bioinformatics and in vitro analyses revealed that SLC2A3 might promote GC progression by upregulating glycolysis reprogramming. Specifically, we demonstrated that SLC2A3 could activate STAT3 signaling pathways. Critical ability of SLC2A3 underlying these activities is the promoting immune infiltration and skewing TAM polarization to the M2-like phenotype. Therefore, our study provides insights into understanding the potential role of SLC2A3 in tumor immunology and its use as a GC biomarker.

Recent studies have revealed that SLC2A3 acts as a transporter with a high affinity for glucose and a high calculated glucose turnover rate in several malignant tumor tissues, including GC [28]. Positive SLC2A3 staining results have been reported in several malignant tumor tissues, suggesting that SLC2A3 may participate in facilitating glucose uptake in tumors with intense glucose requirements [29]. However, most studies have focused on SLC2A1, and there is very limited knowledge of the role of SLC2A3 in GC. In this study, we examined the relationship between SLC2A family expression levels in GC using GEPIA. The database revealed that higher SLC2A3 and SLC2A6 expression were positively correlated with poorer OS and DFS in patients with GC. Therefore, we considered that SLC2A3 and SLC2A6 were important oncogenes that influenced the progress of GC. A previous study described that SLC2A6 expressed in very low levels in GC and did not show any prognostic relevance and that SLC2A3 expression in GC was linked to clinical and pathological parameters [23]. Hence, we selected SLC2A3 as the target of our research. The RNA-seq data of multiple malignancies in TCGA showed that SLC2A3 expression differed in cancer and corresponding normal tissues in a variety of cancers. Although SLC2A3 was more highly expressed in GC cancer samples than in normal tissues, when analyzed TIMER and GEPIA database analyses indicated that these differences were not significant. However, we tested the relationship between SLC2A3 expression and the OS and DFS of patients with GC in our center and found significant differences. In this study, positive SLC2A3 staining correlated with poor prognosis, which is consistent with the findings of previous investigations [20]. Although the mechanism underlying SLC2A3 regulation remains to be determined, these findings strongly suggest that SLC2A3 is a useful prognostic biomarker for GC.

SLC2A proteins facilitate glucose influx into cancer cells which is necessary for cancer cell proliferation [30]. Cancer cells prefer the anaerobic breakdown of glucose for energy rather than mitochondrial oxidative phosphorylation [31]. The Warburg effect, the most common metabolic phenotype in cancer cells, is closely correlated with cancer cell proliferation, metastasis, and drug resistance [32]. As the roles of SLC2A3 in GC remained obscure, we sought to delineate the characters of SLC2A3 in GC tumorigenesis. TIMER and TCGA database indicated that SLC2A3 expression had a linear relationship with the expression of glycolysis enzymes. Here, we demonstrated that SLC2A3 could enhance glycolysis, followed by upregulation of the downstream glycolytic genes SLC2A1, LDHA, HK2, and PKM2 in GC cells. This then leads to an increased glycolytic phenotype and dependence on glucose. Impressively, cells overexpressing SLC2A3 were highly addicted to glucose and increased glycolysis led to rapid cell growth and proliferation in vitro. Whether altered glucose metabolism could modulate malignant phenotype and signaling was not known, nor was the mechanism underlying these observations. Furthermore, our data showed that the STAT3 signaling pathway was activated upon overexpression of SLC2A3. Mechanistically, STAT3 phosphorylation levels were reduced when SLC2A3 expression was knocked down in GC cells. Our findings fill a fundamental gap in our current understanding of the mechanism by which glucose metabolism is involved in cancer. Herein, these results suggest that SLC2A3 promotes GC cell proliferation by inducing glycolysis reprogramming and STAT3 signaling pathway activation.

With the renewed interest in glucose metabolism, researchers have realized that increased glycolysis activity a major consequence of certain oncogenic drivers. Recent findings suggest that intratumoral mechanisms of metabolite communication act symbiotically to support tumor metabolism, maintenance, and growth, or competitively to impair antitumor immunity [23]. TAMs, and the M2-polarized TAMs in particular, have been shown to promote tumorigenesis and drug resistance [33]. The increased abundance of tumor-infiltrating M2 TAMs has been correlated with poor prognosis in various human cancers [34]. Modification and/or disruption of molecular communication between cancer cells and their microenvironment represents an important milieu for intervention development [35]. At the protein level, SLC2A3 is also controlled through membrane localization, which occurs during immune cell activation and could be relevant for its role in the TME [30]. Here, we first demonstrated that SLC2A3 is positively correlated with the expression of the M2 subtype macrophage marker. Our research showed that SLC2A3 is an important oncogene that changes the GC microenvironment by increasing M2 macrophage infiltration. This is likely caused by tumor mediated immune cell recruitment by diverse chemokines that are secreted by tumor cells due to activation of relative signaling in the TME. The composition of immune infiltrates in tumors can also be shaped by nutrient availability in the TME [36]. Malignant cells can deprive the TME of glucose, thus blocking effective anticancer immunity, as glucose is used by macrophages to support their effector functions [37]. Another nutrient, lactate, acts as a metabolic substrate and is secreted to the extracellular microenvironment by cancer cells [38]. Consistent with the results described here, lactate has been shown to increase M2 markers in macrophages, and to favor tumor growth by polarizing macrophages to an M2-like state [39]. Hence, additional experiments will also be needed to determine exactly how SLC2A3 affects macrophage differentiation.

We have postulated that SLC2A3 might regulate the progression of GC by promoting glycolysis reprogramming, activating the STAT3 signaling pathway, and increasing tumor microenvironment macrophage infiltration and M2 subtype transition (Fig. 6c). Hence, SLC2A3 suggest a potential target of additional chemotherapeutic agents for the treatment of GC. However, for anticancer treatment, there should be careful consideration about the potential undesirable impact of blocking SLC2A3 in cells or tissues that express it and need them for physiological glucose homeostasis. Studies have demonstrated that SLC2A3 is a neuronal glucose transporter which is found predominantly in the axons and dendrites [40]. Fortunately, key organs in the body such as the brain can use ketone bodies as a substitute for glucose [41, 42]. Therefore, SLC2A3 inhibition should not result in significant energy shortage for these vital organs. Up to date, no specific SLC2A3 inhibitor has been discovered [30]. Some DNA-damaging anticancer agents including adriamycin, camptothecin and etoposide were reported to induce cancer cell death by reducing SLC2A3 expression in HeLa cells [43]. Hence, further basic science and clinical investigation are warranted. Importantly, improvement in inhibitor’s efficacy (IC50), selectivity of the target, and identification of therapeutic windows while taking cancers’ specific genotype and phenotype into account, are needed for SLC2A3 inhibitors to become effective anti-cancer therapeutics.

## Materials and methods

### Survival data acquisition

The GC patients’ survival data was downloaded from the GEPIA website (https://gepia.cancer-pku.cn). The dataset is an interactive web that includes 9736 tumors and 8587 normal samples from TCGA and the GTEx projects, which analyze the RNA sequencing expression [44]. Another survival data was downloaded from TIMER (https://cistrome.shinyapps.io/timer/). Our centers’ survival data was obtained by our database that contained follow-up data. The detailed clinicopathological parameters were showed in Tables 1 and 2.

### Cell culture and treatment

MKN45, SNU216 and THP-1 monocytes were purchased from ATCC and maintained as described. Cells were routinely cultured and maintained in RPMI 1640 (Gibco) supplemented with 10% fetal bovine serum (Gibco) and antibiotics (Gibco) according to the ATCC protocols. The plasmid or small interfering RNAs (siRNAs, 100 pmol) against human SLC2A3 were transfected into the MKN45 cells or SNU216 cells using Lipofectamine 3000 reagent (Thermo Scientific Dharmacon Inc., USA), while non-specific plasmid or siRNA was used as negative controls. Three siRNA were designed to knock down SLC2A3 expression and the most effective one—SLC2A3#siRNA#2—was used for further examination (Additional file 1: Figure S3a). The oligonucleotide sequences used in this study were displayed in Additional file 1: Table S1. Lentiviral vectors plasmids were constructed by GENECHEM Biotech at Shanghai, China (https://genechem.bioon.com.cn/). Flag-tagged SLC2A3-overexpressed vectors (SLC2A3-LV) and control vectors (Control-LV) were transfected into SNU216 cells to generate cells with stable overexpression of SLC2A3.

### Western blotting analysis

Protein from cells was separated by SDS-PAGE and transferred to PVDF membranes as described. The following primary antibodies were used for western blots: the rabbit β-Actin antibody, SLC2A3 antibody, GPI, HK2, LDHA, and SLC16A1 were used with the concentration of 1:500, Affinity. Cell signal pathway antibodies were purchased from CST group with a concentration of 1:1000, including STAT3, phosphorylated-STAT3.

### Co-IP assay

Total proteins were extracted with cell lysis buffer supplemented with protease inhibitor and phosphatase inhibitor. Lysate (100 μg protein) was incubated with anti-SLC2A3 (1:50, Abcam), anti-p-STAT3 (Tyr705) (1:50, Affinity), or IgG (as a negative control, 1:1000, Cell Signaling Technology) at 4 °C overnight. Then the protein–antibody complex was incubated with protein A/G magnetic beads for 4–6 h at 4 °C. Immunoprecipitation was then collected by centrifugation at 1500 RPM for 15 min at 4 °C and washed the beads complex four times with PBS. After the final wash, protein A/G magnetic beads eluted by boiling 5 min in 100 °C with 2× protein loading buffer before western blot.

### RNA isolation and real-time quantitative PCR analysis

Total RNA from cells was extracted using the Trizol reagent, following by reverse transcription for purified cDNA templates. A SYBR Premix Ex Taq II Kit (Takara) was used to perform real-time RT-PCR in the presence of oligo dT primers (Sangon) according to the manufacturer’s recommendation. The mRNA expression was normalized to β-Actin.

### Immunohistochemistry analysis

As previously described, immunohistochemistry (IHC) was performed to investigate protein expression in tissue. Tumor samples were obtained from the Department of General Surgery, NanFang Hospital, Southern Medical University. The sections of IHC were then incubated with antibodies against SLC2A3 (1:50, Affinity). Intensity of staining of cancer cells was scored as follows: 0 (no staining), 1 (weakly staining, light yellow), 2 (moderately staining, yellow brown), and 3 (strongly staining, brown). An intensity score of ≥ 2 was considered as overexpression, whereas < 2 in the intensity score was regarded as low expression. The discrepancies (< 5%) were resolved by simultaneous reevaluation. All evaluation was analyzed by three independent observers using the same light microscope.

### Proliferation assay

GC cells were transfected with SLC2A3 siRNA and negative control siRNA, and the CCK8 (Dojindo) proliferation assay was performed according to the manufacturer’s instructions for the indicated time.

### Cell migration assay

Transwell chamber migration assay was measured using a transwell chamber with 8 μm filter inserts (Corning). 5 × 10^4^ cells were mixed with 0.2 ml of serum-free medium and seeded to the upper chambers of transwell plates (Corning) as described [45]. In the lower chamber, 0.6 ml of medium with 10% FBS was added to promote cell movement through the pores of the membrane. The inside of the inserts was cleaned thoroughly with a cotton swab, and cells which had migrated through the porous membrane were fixed with a methanol solution for 15 min, and Giemsa staining was performed. The numbers of cells in 4 randomly selected microscope fields were determined.

### Extracellular acidification rate and basal oxygen consumption rate

Oxygen consumption rates (OCR) and extracellular acidification rates (ECAR) were measured using an XF24 extracellular analyzer (Seahorse Bioscience). A 24-well cell culture microplate was coated with Corning^®^ Cell-Tak™ Cell and Tissue Adhesive (Corning Incorporated) to allow adhesion of suspended cells. After calibration of the analyzer, sequential compound injections, including oligomycin A, carbonyl-cyanide p-trifluoromethoxyphenylhydrazone (FCCP), antimycin A and rotenone, were applied on the microplate to test mitochondrial respiration. Sequential compound injections, including glucose, oligomycin A and 2-DG, were applied to test glycolytic activity.

### Lactate production and glucose assay

To measure lactate production or mount of glucose, 1 × 10^5^ cells per well were seeded in 24-well plates in triplicate for 24 h, then the medium was refreshed with RPMI1640 containing 1 mM glucose overnight. The next day, culture medium was collected for measurement of lactate and glucose concentrations as determined by glucose (GO) assay kit (Sigma) and lactate assay kit (Biovision). Lactate production and mount of glucose were normalized by cell numbers.

### Glucose uptake assay

2-NBDG (Life Technologies) was used as a glucose tracer. Briefly, 1 × 10^5^ cells per well were seeded in 6-well plates in quadruplicate and incubated overnight at 37 °C with 5% CO_2_. The next day, cells were starved for glucose for 4 h, then one well was incubated with RPMI1640 medium with 25 μM glucose. As a negative control, and the remaining three wells were cultivated with 25 μM 2-NBDG for 2 h. After incubation, cells were digested and washed twice with PBS. The mean fluorescence intensity of cells was measured by flow cytometry with excitation light at 488 nm.

### Chromatin immunoprecipitation (ChIP) assay

ChIP experiments were performed according to the protocol of Chromatin Immunoprecipitation kit (BersinBio). Immunoprecipitation reactions were performed with antibodies against p-STAT3 or with IgG used as a negative control. Purified DNA was then suspended for following qRT-PCR analysis. PCR products were then run on 2.5% agarose gels and visualized with ethidium bromide. Relative chromatin enrichment was calculated as the amount of amplified DNA normalized to input and relative to values obtained after normal IgG immunoprecipitation.

### Tumor growth assay

All animal experiments were approved by the Institutional Review Board of Nanfang Hospital of Southern Medical University. MKN45 cells or SNU216 cells were suspended in 100 μl PBS at a final concentration of 1 × 10^6^ cells and implanted subcutaneously into the flanks of 4- to 6-week-old male BALB/c nude mice (Laboratory Animal Unit, Southern Medical University, China). The sizes of the resulting tumors were measured weekly. Tumor volumes were calculated as follows: total tumor volume (mm^3^) = (Length × Width^2^)/2, where Length is the longest length.

### AAV-GFP construction and intratumor injection

SLC2A3 knockdown and control recombinant Adeno-associated virus-green fluorescence protein vectors (AAV-GFP) were constructed (GENECHEM Biotech). The administration procedures were performed according to previous studies. Briefly, 1 × 10^9^ physical particles of AAV in 100 μl of PBS were injected into the tumor of nude mice.

### Statistical analysis

Data were analyzed using the student’s t-test or one-way analysis of variance, followed by the Student–Newman–Keuls test using SPSS v20.0 statistical software (SPSS, Inc. Chicago, IL, USA) and the results are expressed as the mean ± standard deviation (SD). A two-tailed probability (ρ)-value of < 0.05 was considered statistically significant.

## Supplementary information

**Additional file 1 Figure S1.** Kaplan–Meier survival curves comparing OS (a–m) and DFS (n–z) between the high and low expression of SLC2A family members in gastric cancer. **Figure S2. **TIMER database analyzed the expression of SLC2A3 in pan-cancer. **Figure S3.** (a) Knockdown effectiveness of four siRNAs were tested by western blot. (b) The effects of SLC2A3 perturbation on other GLUT isoforms and HMIT were assessed by RT-PCR. **Figure S4. **The photo captured by JASPAR to predict the potential binding site in SLC2A3 promoter region by transcriptional factor STAT3. **Table S1.** Primers and RNA sequences used in this study.

## Data Availability

All data generated or analyzed during this study are included in this article.
