# Supplementary material for: SLC2A3 promotes macrophage infiltration by glycolysis reprogramming in gastric cancer
Source: Cancer Cell Int. 2020 Oct 12;20:503. doi: 10.1186/s12935-020-01599-9 (PMC7552479; doi:10.1186/s12935-020-01599-9)
Supplement: Supplementary file 1 — Additional file 1 Figure S1. Kaplan–Meier survival curves comparing OS (a–m) and DFS (n–z) between the high and low expression of SLC2A family members in gastric cancer. Figure S2. TIMER database analyzed the expression of SLC2A3 in pan-cancer. Figure S3. (a) Knockdown effectiveness of four siRNAs were tested by western blot. (b) The effects of SLC2A3 perturbation on other GLUT isoforms and HMIT were assessed by RT-PCR. Figure S4. The photo captured by JASPAR to predict the potential binding site in SLC2A3 promoter region by transcriptional factor STAT3. Table S1. Primers and RNA sequences used in this study. [file 12935_2020_1599_MOESM1_ESM.docx]

**SLC2A3 is a prognostic biomarker correlated with glycolysis reprogramming and macrophage infiltration in gastric cancer**

**
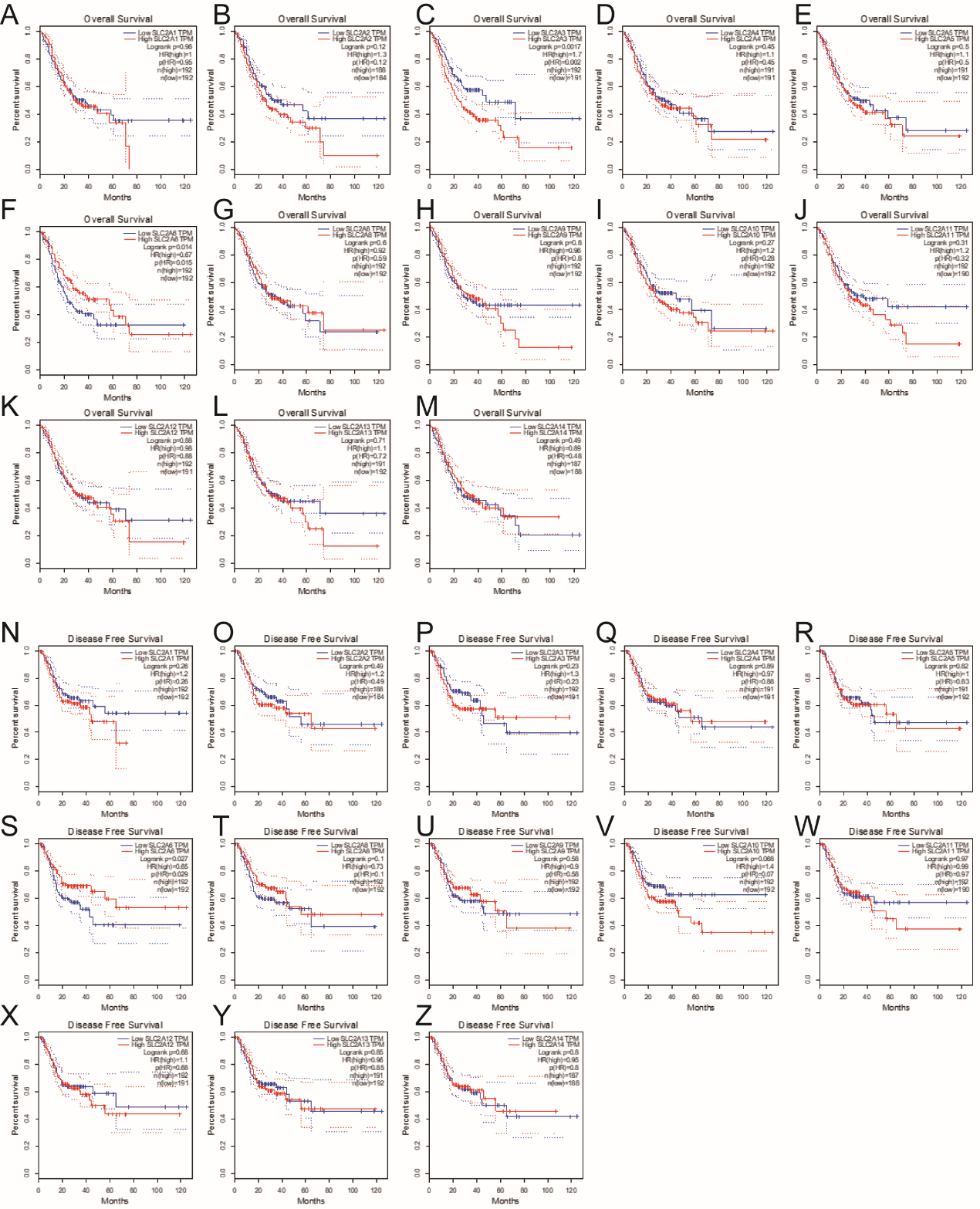
ADDITIONAL INFORMATION**

**Figure S1** Kaplan-Meier survival curves comparing OS (a-m) and DFS (n-z) between the high and low expression of SLC2A family members in gastric cancer. (a, n) OS survival curves (n(high)=192, n(low)=192) and DFS survival curves of gastric cancer by SLC2A1 expression (n(high)=192, n(low)=192). (b, o) OS survival curves (n(high)=188, n(low)=164) and DFS survival curves of gastric cancer by SLC2A2 expression (n(high)=188, n(low)=164). (c, p) OS survival curves (n(high)=192, n(low)=191) and DFS survival curves of gastric cancer by SLC2A3 expression (n(high)=192, n(low)=191). (d, q) OS survival curves and DFS survival curves of gastric cancer by SLC2A4 expression (n(high)=191, n(low)=191). (e, r) OS survival curves (n(high)=191, n(low)=192) and DFS survival curves of gastric cancer by SLC2A5 expression (n(high)=191, n(low)=192). (f, s) OS survival curves (n(high)=192, n(low)=192) and DFS survival curves of gastric cancer by SLC2A6 expression (n(high)=192, n(low)=192). (g, t) OS survival curves (n(high)=192, n(low)=192) and DFS survival curves of gastric cancer by SLC2A8 expression (n(high)=192, n(low)=192). (h, u) OS survival curves (n(high)=192, n(low)=192) and DFS survival curves of gastric cancer by SLC2A9 expression (n(high)=192, n(low)=192). (i, v) OS survival curves (n(high)=192, n(low)=192) and DFS survival curves of gastric cancer by SLC2A10 expression (n(high)=192, n(low)=192). (j. w) OS survival curves (n(high)=192, n(low)=190) and DFS survival curves of gastric cancer by SLC2A11 expression (n(high)=192, n(low)=190). (k, x) OS survival curves (n(high)=192, n(low)=191) and DFS survival curves of gastric cancer by SLC2A12 expression (n(high)=192, n(low)=191). (l, y) OS survival curves (n(high)=191, n(low)=192) and DFS survival curves of gastric cancer by SLC2A13 expression (n(high)=191, n(low)=192). (m, z) OS survival curves (n(high)=187, n(low)=188) and DFS survival curves of gastric cancer by SLC2A14 expression (n(high)=187, n(low)=188).


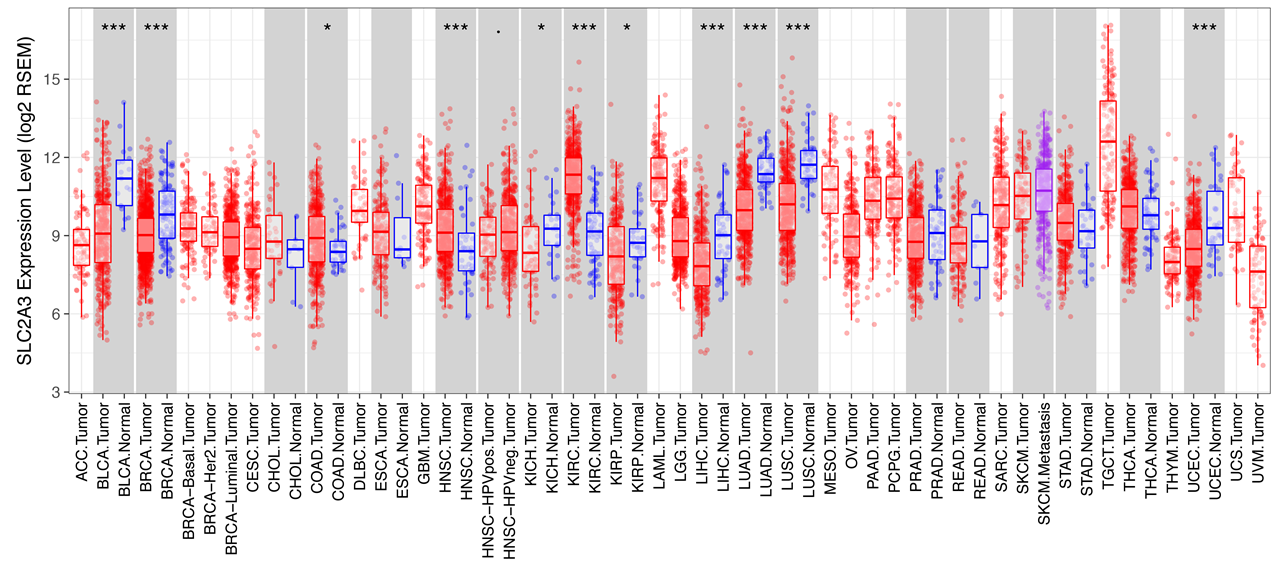


**Figure S2** TIMER database analyzed the expression of SLC2A3 in pan-cancer.

**
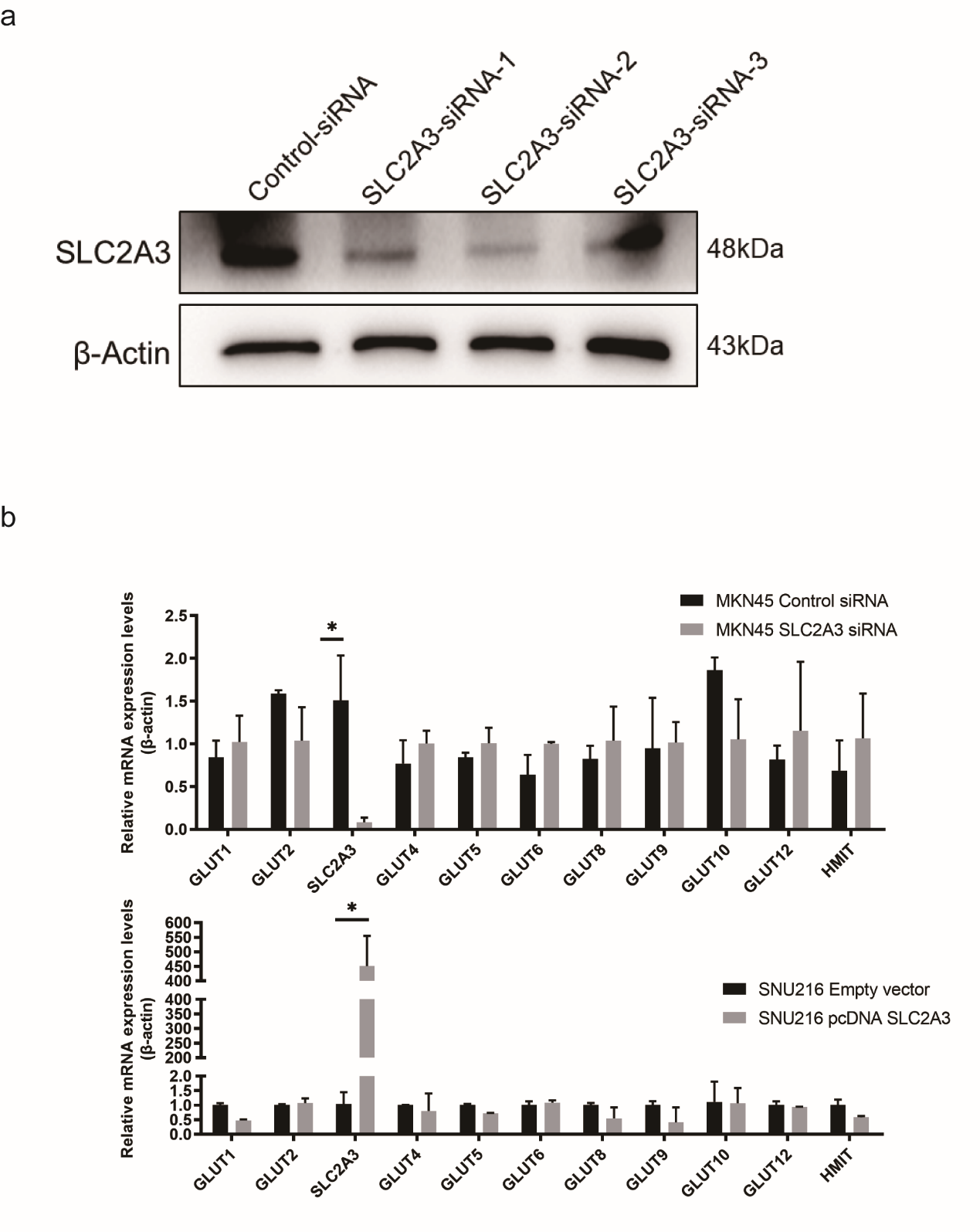
**

**Figure S3** (a) Knockdown effectiveness of four siRNAs were tested by western blot. (b)The effects of SLC2A3 perturbation on other GLUT isoforms and HMIT were assessed by RT-PCR.

**
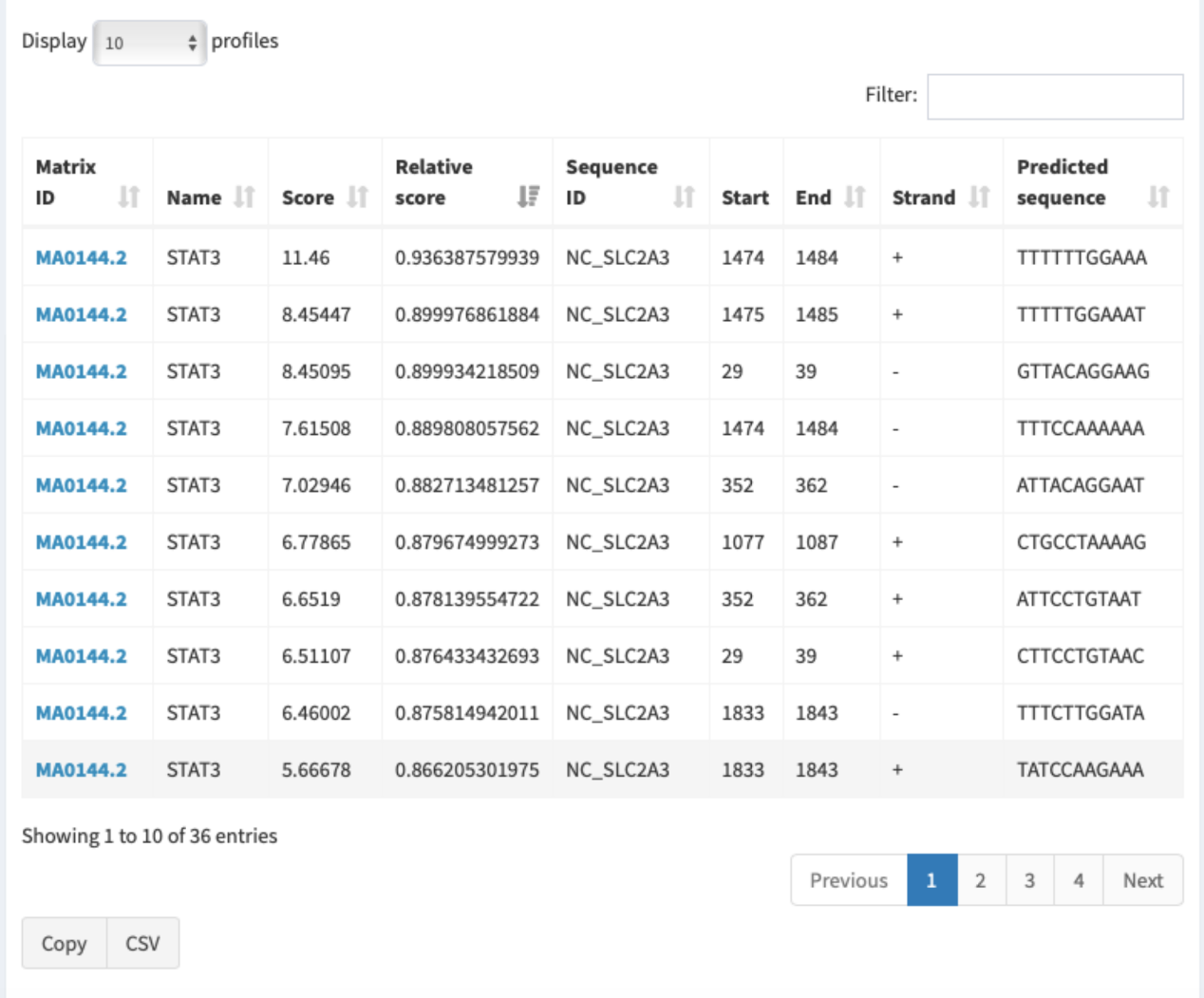
Figure S4** The photo captured by JASPAR to predict the potential binding site in SLC2A3 promoter region by transcriptional factor STAT3.

**Table S1** Primers and RNA sequences used in this study.

| GLUT1 |  |
| --- | --- |
| Forward | TTGCAGGCTTCTCCAACTGGAC |
| Reverse | CAGAACCAGGAGCACAGTGAAG |
|  |  |
| GLUT3 |  |
| Forward | TGCCTTTGGCACTCTCAACCAG |
| Reverse | GCCATAGCTCTTCAGACCCAAG |
|  |  |
| HK2 |  |
| Forward | GAGTTTGACCTGGATGTGGTTGC |
| Reverse | CCTCCATGTAGCAGGCATTGCT |
|  |  |
| GPI |  |
| Forward | CACTGACCACTACTTTCTGCGC |
| Reverse | CTGGTGTGGAACAAGCGATCTG |
|  |  |
| PFKFB3 |  |
| Forward | TCATCGAGTCGGTCTGTGACGA |
| Reverse | CATGGCTTCTGCTGAGTTGCAG |
|  |  |
| PFKFB4 |  |
| Forward | GATCCTGAGGTCATAGCTGCCA |
| Reverse | CTATCCAGGTCCTCATCTAGCG |
|  |  |
| PFKL |  |
| Forward | AAGAAGTAGGCTGGCACGACGT |
| Reverse | GCGGATGTTCTCCACAATGGAC |
|  |  |
| PFKP |  |
| Forward | AGGCAGTCATCGCCTTGCTAGA |
| Reverse | ATCGCCTTCTGCACATCCTGAG |
|  |  |
| ALDOA |  |
| Forward | GACACTCTACCAGAAGGCGGAT |
| Reverse | GGTGGTAGTCTCGCCATTTGTC |
|  |  |
| TPI1 |  |
| Forward | CGAGCAGACAAAGGTCATCGCA |
| Reverse | TCGGAGCTTCTCGTGTACTTCC |
|  |  |
| GAPDH |  |
| Forward | GTCTCCTCTGACTTCAACAGCG |
| Reverse | ACCACCCTGTTGCTGTAGCCAA |
|  |  |
| PGK1 |  |
| Forward | CCGCTTTCATGTGGAGGAAGAAG |
| Reverse | CTCTGTGAGCAGTGCCAAAAGC |
|  |  |
| PGAM1 |  |
| Forward | GCTCTGCCCTTCTGGAATGAAG |
| Reverse | ATACCAGTCGGCAGGTTCAGCT |
|  |  |
| SLC16A3 |  |
| Forward | CCACAAGTTCTCCAGTGCCATTG |
| Reverse | CGCCAGGATGAACACGTACATG |
|  |  |
| SLC16A1 |  |
| Forward | TTGTTGGTGGCTGCTTGTCAGG |
| Reverse | TCATGGTCAGAGCTGGATTCAAG |
|  |  |
| H6PD |  |
| Forward | GGTGGACCATTACTTAGGCAAGC |
| Reverse | CTTCAGCATCCACGGTCTCTTTC |
|  |  |
| G6PD |  |
| Forward | CTGTTCCGTGAGGACCAGATCT |
| Reverse | TGAAGGTGAGGATAACGCAGGC |
|  |  |
| LDHB |  |
| Forward | GGACAAGTTGGTATGGCGTGTG |
| Reverse | AAGCTCCCATGCTGCAGATCCA |
|  |  |
| LDHA |  |
| Forward | GGATCTCCAACATGGCAGCCTT |
| Reverse | AGACGGCTTTCTCCCTCTTGCT |
|  |  |
| PKM |  |
| Forward | ATGGCTGACACATTCCTGGAGC |
| Reverse | CCTTCAACGTCTCCACTGATCG |
|  |  |
| ENO1 |  |
| Forward | AGTCAACCAGATTGGCTCCGTG |
| Reverse | CACAACCAGGTCAGCGATGAAG |
|  |  |
| PGAM4 |  |
| Forward | CGCTACGAGATGCTGGCTATGA |
| Reverse | CCATAGTGCCGCTCATTGAGGC |
|  |  |
| iNOS |  |
| Forward | GCTCTACACCTCCAATGTGACC |
| Reverse | CTGCCGAGATTTGAGCCTCATG |
|  |  |
| TNFa |  |
| Forward | CTCTTCTGCCTGCTGCACTTTG |
| Reverse | ATGGGCTACAGGCTTGTCACTC |
|  |  |
| CD80 |  |
| Forward | CTCTTGGTGCTGGCTGGTCTTT |
| Reverse | GCCAGTAGATGCGAGTTTGTGC |
|  |  |
| CD86 |  |
| Forward | CCATCAGCTTGTCTGTTTCATTCC |
| Reverse | GCTGTAATCCAAGGAATGTGGTC |
|  |  |
| IL4 |  |
| Forward | CCGTAACAGACATCTTTGCTGCC |
| Reverse | GAGTGTCCTTCTCATGGTGGCT |
|  |  |
| IL13 |  |
| Forward | ACGGTCATTGCTCTCACTTGCC |
| Reverse | CTGTCAGGTTGATGCTCCATACC |
|  |  |
| CD163 |  |
| Forward | CCAGAAGGAACTTGTAGCCACAG |
| Reverse | CAGGCACCAAGCGTTTTGAGCT |
|  |  |
| CD206 |  |
| Forward | AGCCAACACCAGCTCCTCAAGA |
| Reverse | CAAAACGCTCGCGCATTGTCCA |
|  |  |
| ARG1 |  |
| Forward | TCATCTGGGTGGATGCTCACAC |
| Reverse | GAGAATCCTGGCACATCGGGAA |
|  |  |
| **siRNAs** | **sense (5'-3')** |
| SLC2A3-siRNA-1 | GCAUCGUUGUUGGAAUUCUTT |
| SLC2A3-siRNA-2 | GUAGCUAAGUCGGUUGAAATT |
| SLC2A3-siRNA-3 | GCUCUUUCCAAUUUGGCUATT |
| Control-siRNA | UUCUCCGAACGUGUCACGUTT |
